# Advances and optimization strategies in bacteriophage therapy for treating inflammatory bowel disease

**DOI:** 10.3389/fimmu.2024.1398652

**Published:** 2024-05-08

**Authors:** Yang Li, Xiao-meng Li, Hao-yu Duan, Kai-di Yang, Jun-feng Ye

**Affiliations:** ^1^ General Surgery Center, First Hospital of Jilin University, Changchun, Jilin, China; ^2^ Department of Rehabilitation, School of Nursing, Jilin University, Changchun, China

**Keywords:** inflammatory bowel disease (IBD) treatment, gut microbiota, bacteriophage therapy, microbiome modulation, encapsulation and targeted delivery

## Abstract

In the advancement of Inflammatory Bowel Disease (IBD) treatment, existing therapeutic methods exhibit limitations; they do not offer a complete cure for IBD and can trigger adverse side effects. Consequently, the exploration of novel therapies and multifaceted treatment strategies provides patients with a broader range of options. Within the framework of IBD, gut microbiota plays a pivotal role in disease onset through diverse mechanisms. Bacteriophages, as natural microbial regulators, demonstrate remarkable specificity by accurately identifying and eliminating specific pathogens, thus holding therapeutic promise. Although clinical trials have affirmed the safety of phage therapy, its efficacy is prone to external influences during storage and transport, which may affect its infectivity and regulatory roles within the microbiota. Improving the stability and precise dosage control of bacteriophages—ensuring robustness in storage and transport, consistent dosing, and targeted delivery to infection sites—is crucial. This review thoroughly explores the latest developments in IBD treatment and its inherent challenges, focusing on the interaction between the microbiota and bacteriophages. It highlights bacteriophages’ potential as microbiome modulators in IBD treatment, offering detailed insights into research on bacteriophage encapsulation and targeted delivery mechanisms. Particular attention is paid to the functionality of various carrier systems, especially regarding their protective properties and ability for colon-specific delivery. This review aims to provide a theoretical foundation for using bacteriophages as microbiome modulators in IBD treatment, paving the way for enhanced regulation of the intestinal microbiota.

## 1 Introduction

Inflammatory Bowel Disease (IBD) is a collection of autoimmune disorders impacting the gastrointestinal tract, influenced by the gut microbiota. Bacteriophages, viruses that infect specific bacteria, play a role in modulating these microbial communities and may offer a strategy to combat IBD-related pathogens. Although still in early stages, phage therapy shows promise, with recent advancements focusing on optimizing these treatments for IBD. Specially formulated phage therapies targeting specific pathogenic strains could minimize adverse effects on beneficial gut bacteria, yet challenges in phage delivery and specificity remain. Antibiotics, while effective, indiscriminately kill gut bacteria and contribute to dysbiosis and resistance issues (1). Traditional methods like probiotics and fecal microbiota transplantation face hurdles like poor colonization and risk of transferring harmful agents (2, 3). Advances in gut microbiology have led to innovative strategies such as targeted bacteriophage delivery systems for the colon (4), using stabilizers like Eudragit FS30D, which have shown promising stability and efficacy (5). Therefore, bacteriophages are increasingly recognized as potential tools for precisely modulating the gut microbiota and addressing intestinal disorders. Bacteriophages face challenges such as high temperatures, low pH, and digestive enzymes, which can diminish their effectiveness during storage and gastrointestinal transit (6–8). Encapsulation has proven a valuable method to enhance bacteriophage stability, ensuring their viability under these conditions. Ma et al. demonstrated that encapsulating bacteriophage Felix O1 in alginate-chitosan microspheres significantly improved its survival in simulated porcine gastrointestinal environments, indicating potential for effective therapeutic delivery to the intestines (9). This paper reviews recent progress in IBD treatment, examining the interaction between the microbiome and bacteriophages, and discussing the mechanisms and potential of bacteriophage-mediated modulation of gut microbiota. It covers the influence of preparation, storage, and delivery on bacteriophage vitality and provides a summary of diverse encapsulation materials used for bacteriophage delivery. The conclusion proposes future directions for bacteriophage delivery strategies in manipulating the gut microbiota.

## 2 Treatment methods for IBD

IBD is a chronic autoimmune inflammatory condition affecting the gastrointestinal tract and extraintestinal organs. This disease encompasses various clinical and histological variations, including ulcerative colitis (UC), Crohn’s disease (CD), and indeterminate colitis. The treatment strategies for IBD have continuously evolved and been explored in recent years (**Table 1**).

**Table 1:** Overview of IBD treatment methods. [Adapted from (10)].

Treatment	Medications	Mechanism	Clinical Applications	
Drug Therapy	Aminosalicylates Antibiotics Corticosteroids	Alleviate inflammation; Inhibit abnormal proliferation; Regulate immune dysregulation	5-ASA offers specific relief for Crohn's patients; Corticosteroids are commonly prescribed	
Immunomodulators	Thiopurines Cyclosporine	Regulate the immune system	Thiopurine analogs prevent relapse in ulcerative colitis and Crohn's disease (11); They're commonly used to maintain remission	
Biologics	Infliximab Adalimumab	Reduce inflammation levels; Regulate the immune system	In treating IBD, Infliximab performs exceptionally well; Adalimumab has also been proven effective	
Stem Cell Therapy	Stem cells	Repair damaged tissues; Promote healing; Anti-inflammatory	More research is needed to assess the safety and effectiveness of reconstructing damaged intestinal mucosa	
**Treatment**		**Introduction**	**Clinical Applications**
Fecal Microbiota Transplantation (FMT)		FMT rebuild the gut microbiome, restoring balance, diversity, and treating intestinal and extra-intestinal diseases; effectively relieve conditions such as ulcerative colitis and other IBDs	Balancing the gut microbiome and regulating immunity effectively treats IBD, providing a promising treatment approach; Yet, further research is needed to assess its safety and long-term effects
Targeting Intestinal Epithelial Cells		Targeting intestinal epithelial cells with bacteriophages as a novel immunotherapy for IBD, it exhibits immunomodulatory properties, balancing inflammation&tolerance; promise as a prospective therapy	With potential for treating inflammatory bowel diseases, bacteriophages offer new directions in IBD therapy; Their antimicrobial activity and immunomodulatory properties provide promising avenues for IBD treatment

### 2.1 Drug therapy

Presently, therapeutic drugs for IBD encompass a range of medications, including aminosalicylates, antibiotics such as metronidazole, corticosteroids, among others (12). 5-Aminosalicylate (5-ASA) salts stand out as a common pharmaceutical intervention for treating IBD, with sulfasalazine exhibiting specific alleviative effects in Crohn’s disease patients. The precise mechanism through which aminosalicylate salts ameliorate IBD remains uncertain. 5-ASA may possess antioxidant, anti-proliferative, or pro-apoptotic activities, allowing for localized treatment on the gastrointestinal mucosa (13–15). The potential effectiveness of 5-ASA in producing significant effects through the alleviation of mucosal inflammation has been documented (16–18). Likewise, corticosteroids prove effective in mitigating inflammatory responses, exhibiting immunomodulatory effects and finding extensive applications in treatment (19–21). Glucocorticoids are commonly employed for more severe conditions than those treated with 5-ASA (22), and their administration may be escalated when 5-ASA treatment proves ineffective (23).

### 2.2 Immunomodulators and biologics

Immunomodulators and biologic agents, which possess the capability to regulate the immune system, have found widespread application in alleviating symptoms and sustaining remission (24, 25). Nevertheless, some patients may develop tolerance or adverse reactions to these medications. Given the pivotal role of inflammatory factors in the pathogenesis of IBD, biologic agents endowed with anti-inflammatory efficacy have emerged. Infliximab, the first anti-TNF-α drug, has demonstrated outstanding therapeutic efficacy for IBD (26, 27). Simultaneous adoption of therapeutic drug monitoring (TDM) has been shown to optimize the efficacy of infliximab (28). Furthermore, adalimumab and golimumab have also been confirmed as effective treatments for IBD (29, 30). Vedolizumab, a specific antagonist against the α4/β7 integrin, inhibits the interaction between integrins and MAdCAM-1, effectively blocking the homing of immune cells to intestinal tissues and reducing mucosal inflammation in IBD patients (31). This mechanism could offer new opportunities for modulating macrophage-related processes such as mucosal healing (32). Numerous studies have demonstrated its efficacy in treating IBD; for instance, an analysis of homing receptor expression on T cells in peripheral blood and inflamed mucosa showed that treatment with vedolizumab is associated with a significant expansion of regulatory T cells in peripheral blood without a significant increase in viral infections in the IBD group (33). Therefore, as the only gut-selective biologic that specifically targets the α4β7 gastrointestinal integrin receptor, vedolizumab represents a promising therapeutic option for IBD (34). Ustekinumab, a human IgG antibody targeting the p40 subunit common to both IL-12 and IL-23, operates differently from anti-TNF therapies (35). It inhibits the biological activity of these cytokines by blocking their shared p40 subunit, impacting receptors on T cells, natural killer cells, and antigen-presenting cells (36). Monoclonal antibodies against the IL-12/23 p40 subunit have shown significant therapeutic effects in mouse models of colitis (37, 38), suggesting their potential efficacy in treating related diseases. Tumor necrosis factor-like cytokine 1A (TL1A) is associated with IBD and influences the location and severity of intestinal inflammation and fibrosis, showing increased expression in inflamed intestinal mucosa (39). TL1A is closely linked to mucosal immunity, suggesting that blocking it could have potential benefits for inflammatory diseases involving the mucosal surface, such as IBD and asthma. Moreover, the TL1A-DR3 pathway has a specific association with fibrosis in Crohn’s disease (40). Studies have demonstrated that TL1A antibodies effectively alleviate intestinal inflammation and reverse fibrosis to baseline levels in mouse models (41), highlighting TL1A as a promising target in IBD treatment.

### 2.3 Intestinal microbiota

#### 2.3.1 Faecal microbiota transplantation

Fecal microbiota transplantation (FMT) transfers feces from healthy individuals to recipients to restore gut microbiota (42), first used to treat pseudomembranous enterocolitis (43). Increasing research links IBD to dysregulated gut microbiota (44, 45), with microbial changes causing mucosal immune dysregulation and increasing susceptibility to IBD (46). FMT has proven effective for IBD treatment (47), as shown by randomized trials like Moayyedi et al., which found FMT more effective than placebos in inducing remission in active ulcerative colitis (48). Other studies, including a multicenter trial in Australia, demonstrated high-dose, multi-donor FMT’s ability to induce clinical remission in active UC (49), and a trial with 73 adults showed that anaerobically prepared donor FMT was likelier to induce remission than autologous FMT (50). Recent research robustly supports FMT as a promising therapy for IBD. A key study (NCT03426683) involving 35 IBD patients, split into two groups-one receiving standardized FMT and the other traditional medications-over a year, assessed FMT’s efficacy, durability, and safety, while identifying specific bacteria involved in the process. With more clinical trials underway, FMT is likely to become an established IBD treatment, providing relief for patients not responding to conventional therapies by modulating immune responses and reducing inflammation (51). Meta-analyses also support FMT’s efficacy; for instance, Sudarshan et al.’s review of 53 studies noted better UC remission with more frequent FMT infusions and optimized administration (52). Another meta-analysis showed FMT as a successful treatment for CDI in IBD patients, advocating for more trials and research to confirm its effectiveness (53).

#### 2.3.2 Antibiotic therapy for IBD

IBD is linked to host-microbe interactions and gut microbiome imbalances that promote the colonization of opportunistic pathogens, crucial to the disease’s development (54). Certain gut bacteria exacerbate intestinal inflammation in IBD by releasing antigens or stimulatory factors, with studies indicating that microbial diversity reductions are significantly associated with IBD onset (55). Antibiotics like metronidazole and ciprofloxacin are effective in modulating the gut microbiota and treating IBD (56). A study showed that ciprofloxacin, despite its good tolerability, did not significantly differ from placebos in inducing remission in perianal Crohn’s disease due to its tolerability (57). Additionally, ciprofloxacin demonstrated anti-inflammatory properties in a colitis mouse model (58). However, the broad-spectrum activity of most antibiotics affects both harmful and beneficial bacteria, potentially leading to adverse outcomes when overused (59). Long-term use of antibiotics in IBD must balance efficacy against side effects: metronidazole often has severe adverse effects (60), while ciprofloxacin is well-tolerated but expensive and can cause nausea; combined use can increase side effects significantly (61). Judicious use of antibiotics, adhering to clinical guidelines and minimizing doses and duration, can optimize benefits while reducing risks of resistance and adverse effects.

### 2.4 Targeting intestinal epithelial cells

The intestinal epithelial layer acts as a barrier that separates the organism from the external environment (62). Once there is a disruption in the balance of the gut barrier microbiota, uncontrolled immune reactions may erupt in the intestinal microenvironment. This imbalance can lead to unrestricted microbial growth, resulting in various diseases, including IBD (63). Deficiencies in epithelial lymphocytes, chemokine receptor expression, and pattern recognition receptors can lead to abnormal immune responses, subsequently promoting cell differentiation and increasing inflammation (64). Consequently, bacteriophage targeting of intestinal epithelial cells is being explored as a novel immunotherapeutic approach for treating IBD (65). Górski et al. posit that bacteriophages, beyond their antimicrobial activity, also exhibit immunomodulatory characteristics that could be beneficial in the treatment of IBD.

### 2.5 Treatment limitations

The majority of drugs available for IBD offer symptomatic relief rather than a complete cure. Additionally, a significant proportion of these drugs are associated with severe adverse reactions. Prolonged use of 5-ASA in IBD patients may lead to side effects such as headaches, diarrhea, nausea, and even severe complications like pneumonia, hepatitis, and myocarditis (66). Corticosteroids, while potent and rapid in treating IBD, fail to effectively maintain remission and can induce irreversible complications with long-term use, including cataracts, glaucoma, hypertension, and diabetes (67, 68). While biologics broaden the therapeutic spectrum for IBD, they unfortunately bring about severe complications such as tuberculosis, fungal infections, cancer, and tumors. Beyond the serious adverse reactions of medications, the exorbitant cost of IBD treatment imposes a substantial economic burden on patients. Consequently, the development of safe, effective, and cost-efficient therapeutic drugs for clinical treatment of IBD holds paramount significance.

## 3 Interaction between microbiota and bacteriophages in IBD

### 3.1 Role of bacteriophages in IBD

Increasing evidence in humans suggests that the microbiome plays a crucial role in the pathogenesis of IBD. For instance, clinical trial results (Clinical Trial ID: UMIN 000004123) indicate that inter-individual variations in the gut microbiota may be associated with individual differences in the risk of IBD or other diseases (69). Alterations in the gut microbiota are believed to play a pivotal role in the onset, progression, and severity of the disease. Transferring dysregulated IBD-associated microbiota to healthy mice has been shown to induce intestinal inflammation (70–72). Bacteriophages are considered a part of the gut microbiome and may influence bacterial community structure in various clinical settings. The administration of exogenous bacteriophages may represent a potential strategy for inhibiting IBD-associated pathogenic bacteria (73). Alterations in the community structure of IBD may be influenced by the prolific reproduction of bacteriophages within the intestinal bacterial ecosystem. The specific targeting of pathogenic strains by a tailored combination of bacteriophages holds potential as an eradication therapy for preventing and treating IBD, while minimizing adverse effects on the surrounding bacterial microbiome (**Table 2**).

**Table 2:** Summary of phage applications in IBD treatment and modulation of gut microbiota. [Adapted from (74–77)].

References/ Clinical Trial ID	Trial Content	Summary of Results
(78) (Federici et al., 2022)	In four IBD cohorts (n=537), specific antibiotic-resistant Klebsiella pneumoniae (Kp) strains are closely linked with disease severity; This study aims to develop a soluble phage cocktail targeting both sensitive and resistant Kp strains in IBD, using diverse mechanisms to suppress Kp, curb inflammation, and ease disease severity	Assessments of Kp-targeting phages in artificial human gut and healthy volunteer models demonstrate their adaptability, safety, and efficacy in the lower intestinal tract, influenced by gastric acid; Highlights oral phage therapy's potential to effectively suppress non-communicable pathogens and overcome resistance
(79) (Titécat et al., 2022)	Assessed the in vitro efficacy and specificity of seven lytic cocktail phages (EcoActive™) against 210 clinical AIEC strains and 43 non-E. coli strains; In vivo experiments in healthy and AIEC-infected mice corroborate these findings regarding safety and efficacy	EcoActive cocktail effectively targets 95% of AIEC strains in vitro, sparing 43 non-E. coli commensal strains, unlike traditional antibiotics; Long-term administration of the EcoActive cocktail in healthy mice is safe and does not disrupt gut microbiota balance; Supporting phage therapy to reduce AIEC in IBD intestines
NCT03808103	A double-blind randomized controlled trial was conducted with 30 participants; Oral administration of bacteriophages was employed	Evaluating the safety and effectiveness of intestinal invasive Escherichia coli in patients with inactive Crohn's disease; The aim is to improve the course of Crohn's disease by infecting and killing specific types of bacteria
(80) (Duerkop et al., 2018)	Using sequence-independent methods to select viral allelic genes; Applying quantitative metagenomics to study intestinal bacteriophages in a mouse colitis model	Observed shifts in colitis-associated bacteriophage populations, including changes in specific phages linked to the diseased microbiome host; Overlap between bacteriophage populations in healthy and diseased mice and those in healthy individuals and patients with intestinal diseases
NCT03269617	A randomized placebo-controlled trial was conducted with 43 participants; Participants received an oral bacteriophage mixture	Assessing the impact of bacteriophage mixture on individual gut bacteria; Evaluating changes in metabolic syndrome, inflammatory markers, microbial metabolites, and gastrointestinal discomfort perception
(81) (Sinha et al., 2022)	Using in vivo microbial cross-infection experiment; Tracking the effects of fecal viral-like particles isolated from ulcerative colitis patients and healthy controls on bacterial diversity and the severity of experimental colitis in human microbiota-associated (HMA) mice	Found that several phages were transferred to HMA mice, resulting in therapy-specific changes in the gut virome; Phages as dynamic regulators of gut bacterial communities, as suggested by recent literature

**Figure 1:**
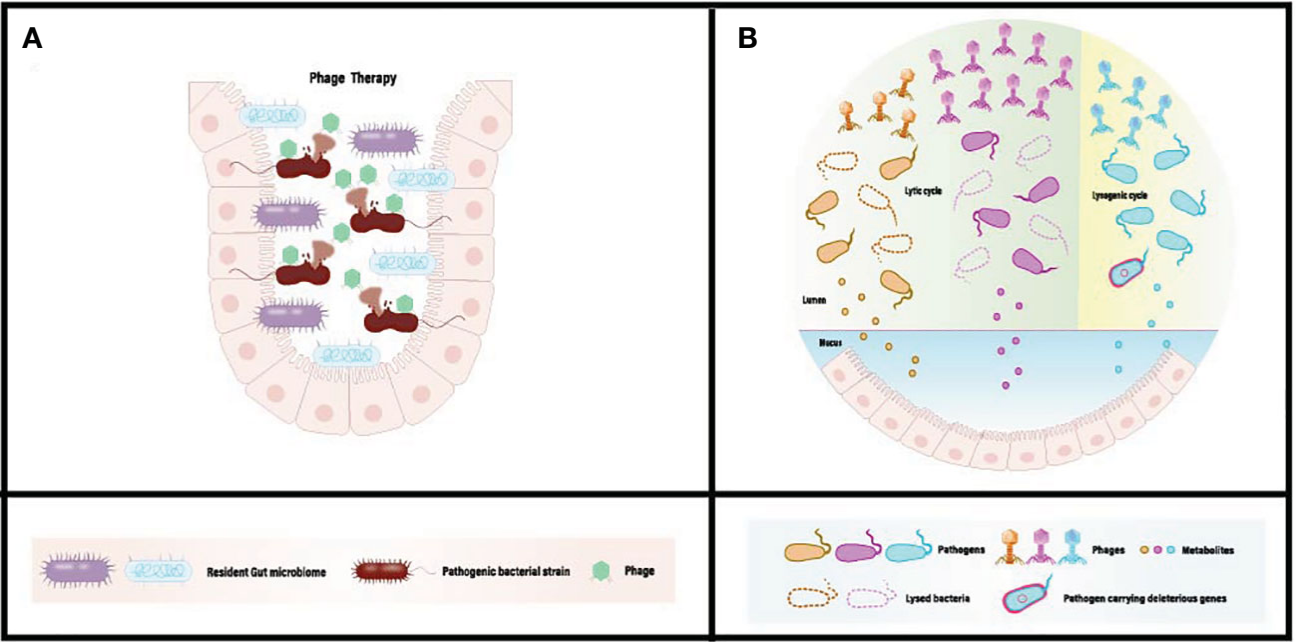
**(A)** Conventional phage therapy targeting gut dysbiosis associated with pathogenic microbiome. **(B)** Administered phages actively diminish the population of pathogenic bacteria, eliminate harmful bacterial genes, and mitigate the absorption of detrimental metabolites *in situ*. [Adapted from (82, 83)].

Bacteriophage-mediated regulation of the gut microbiota is achieved through interactions between bacteria and phages within the colon. Acting as natural predators of bacteria, phages can disrupt or lyse host bacteria through processes such as lytic replication or lysogenic cycles. In cases of gut microbiota dysbiosis, an excess of pathogens or harmful bacteria can produce unwanted metabolites (**Figure 1**). These metabolites are absorbed into the systemic circulation, triggering related symptoms. Upon reaching the colon, bacteriophages can selectively kill host pathogens, reducing adverse metabolites through specific recognition and infection, while minimizing disruption to non-targeted microorganisms (**Figure 1**). Furthermore, the ecological niche of pathogens can be occupied and colonized by symbiotic or probiotic bacteria, restoring gut homeostasis and promoting overall health. In recent research, Lv et al. isolated a strain of Lactobacillus subspecies, Lactobacillus SF, from the feces of healthy infants and conducted a systematic probiotic evaluation. The results demonstrated that Lactobacillus SF could restore gut microbiota balance by increasing the relative abundance of bacteria capable of occupying the same ecological niche as pathogenic bacteria, thereby reducing the survival space for pathogens. This indicates that Lactobacillus SF exhibits excellent resistance to gastric fluids, colonization in the intestine, and strong antibacterial and antioxidant capabilities (84).

### 3.2 Personalized bacteriophage therapy

**Figure 2:**
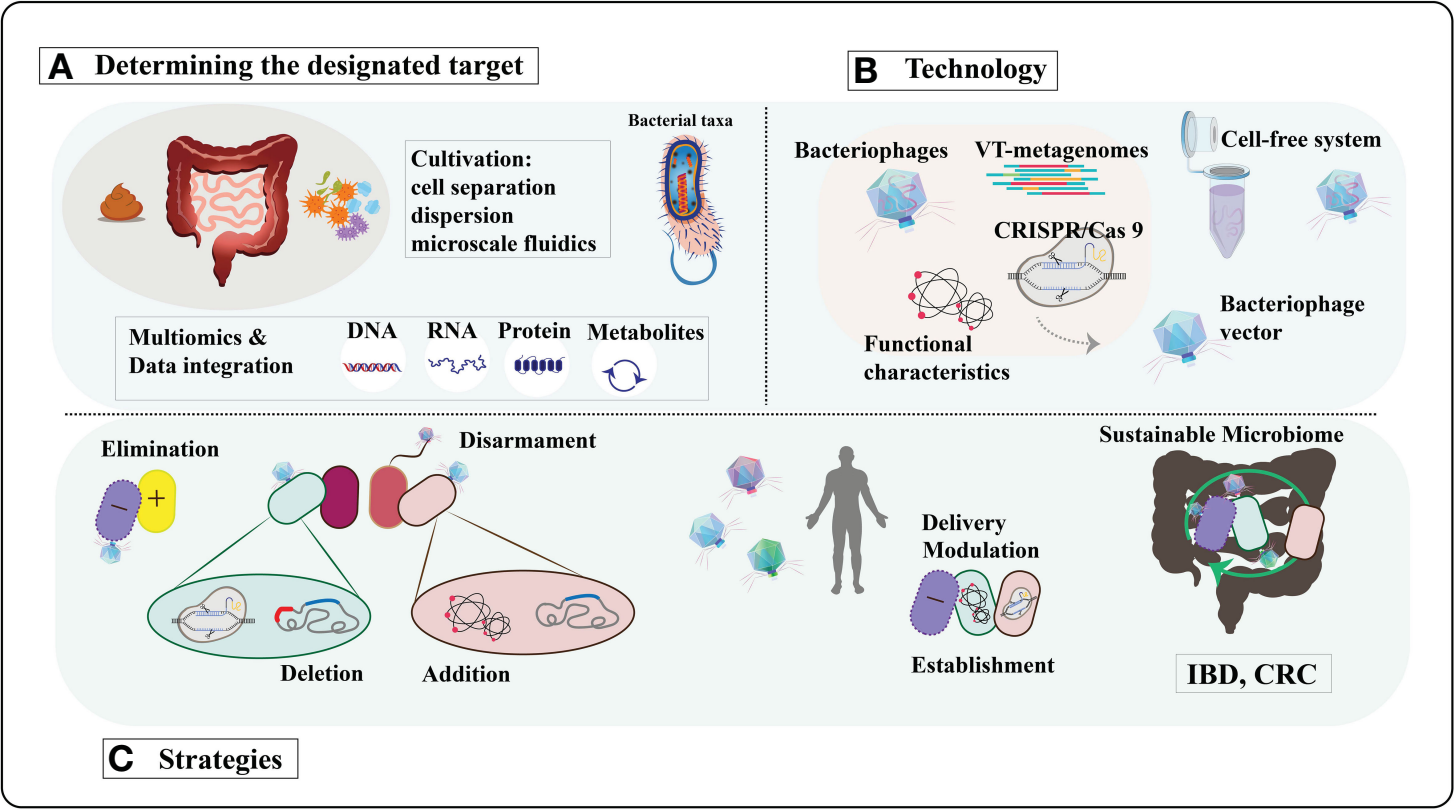
A comprehensive exploration of bacteriophage-derived tools and strategies for precisely modulating the human microbiome. **(A)** Determining the designated target. Bacteriophages will be sourced from diverse environments using cultivation-independent techniques and replicated through cell-free systems or innovative cultivation methods. Identification of bacterial and functional targets will leverage advancements in culturing methods and multi-omics approaches. **(B)** Technology. The isolated phages and phage enzymes will be harnessed to modulate the human microbiota using various technologies. CRISPR/Cas9 will be transported to the target bacteria utilizing modified phage vectors. VT, viral-tagging. **(C)** Strategies. Moreover, to transport specific genes with diverse functions for modifying target bacteria. The ultimate objective is to reinstate a resilient microbiome in diseases associated with dysbiosis, such as inflammatory bowel disease (IBD), colorectal cancer (CRC), and so forth. [Adapted from (95)].

In the human microbiome, bacteriophages can be employed for targeted elimination of specific pathogens without causing harm to beneficial microbial communities (85, 86). With a plethora of phages available, their precise and widespread regulatory capabilities make them an ideal tool for modulating the human microbiome (87). Bacteriophages exhibit high diversity and adaptability, enabling them to thrive in various environments and target different bacterial species (87).Tailoring bacteriophages based on the microbial characteristics of different populations allows for personalized therapeutic interventions and modulation. The low toxicity of bacteriophages in regulating the human microbiome minimizes the risk of severe immune reactions or adverse effects (88, 89). Their self-replication mode enables effective treatment at low doses (90), providing a high level of safety and feasibility in utilizing bacteriophages for human microbiome modulation. In terms of infection mechanisms, bacteriophages can utilize both lytic and lysogenic pathways to infect hosts, broadening their applicability (91, 92). Co-evolution with bacterial hosts enhances their plasticity and survival rates (93, 94). Moreover, bacteriophages can serve as vectors by introducing new functionalities or silencing virulence factors to target bacterial pathogenicity. Bacteriophages possess unique characteristics that position them as precise tools for human microbiome modulation, holding tremendous potential for revolutionary breakthroughs in the regulation and treatment of the human microbiome through further research and development (**Figure 2**).

Viral tagging (VT) technology leverages the specificity of bacteriophages for host bacterial infection, enabling the isolation and purification of bacteriophages by marking and separating host bacteria (96). High-purity bacteriophage samples obtained through VT technology are crucial for microbial community modulation. The use of high-purity bacteriophage samples enhances the effectiveness of microbial community modulation by facilitating accurate assessment and control of the composition and functions of microbial communities. The high purity of bacteriophage samples also aids in reducing unwanted interference from host bacteria or other contaminants, thereby better achieving the desired effects of microbial community modulation. Obtaining high-purity bacteriophage samples through VT technology is a critical prelude to microbial community modulation. Research by Agarwal et al. demonstrates that polymers loaded onto bacteriophages, administered through dry powder inhalation, deposit throughout the lungs, providing active bacteriophages. This ultimately significantly reduces mouse infections and associated inflammation, successfully saving mice from mortality (97). Another study evaluated the effectiveness of bacteriophage therapy in treating methicillin-resistant Staphylococcus aureus-induced mastitis in a mouse model (98).In the mastitis mouse model, treatment with bacteriophage 4086-1 markedly inhibited the proliferation of Staphylococcus aureus in the mammary gland. Concentrations of the inflammatory markers TNF-α and IL-6 were significantly reduced, indicating the effective alleviation of the inflammatory response by bacteriophages.

### 3.3 Prospects and challenges in treatment

The bacterial lysis induced by lytic bacteriophages may lead to toxin contamination and the transfer of virulence genes among gut microbiota (99). In recent years, with the rapid advancements in synthetic biology and sequencing technologies, lysogenic bacteriophages have provided a feasible method for modifying bacterial genes and promoting intestinal homeostasis (100–103). During lysogeny, engineered lysogenic bacteriophages can introduce nucleic acids into the host chromosome and express carried functional genes, thereby enabling precise *in situ* regulation of gene expression in individual microorganisms. For example, the CRISPR/Cas9 system targeting bacterial superantigenicity and cell lysis genes has been integrated into the Staphylococcus aureus lysogenic bacteriophage, resulting in the excision of major virulence genes from the host genome. This has developed into an effective strategy to prevent toxin contamination and virulence gene transfer among gut microbiota (104). However, bacteriophage-mediated therapeutic outcomes are not always effective. In an early clinical trial, despite the dose-dependent detection of T4 bacteriophages in volunteer feces after oral bacteriophage delivery, significant bacteriophage replication and a decrease in total Escherichia coli counts were not observed. Similarly, Sarker et al. found that delivered T4 bacteriophages (108 PFU) failed to proliferate on pathogenic Escherichia coli, and no improvement in diarrhea was observed. One possible cause of bacteriophage replication failure may be the presence of bacterial defense mechanisms, which inhibit bacteriophage invasion and result in treatment failure. It has been reported that anti-CRISPR bacteriophages can collaboratively overcome host bacteria’s CRISPR resistance, rendering the host susceptible to subsequent bacteriophage infections. Therefore, delivering a mixture of bacteriophages targeting the same host (bacteriophage cocktail) could be a potential solution to increase infection likelihood. Another reason for bacteriophage replication failure may be insufficient active bacteriophage numbers in the colon (105). Indeed, bacteriophage viability is affected by various factors during storage and gastrointestinal transport, such as high temperatures and extreme pH values, leading to a significant reduction in bacteriophage titers and subsequent replication and proliferation failure (106, 107). Developing bacteriophage combination-mediated IBD pathogen eradication therapy may require better strain-level bacterial target identification and addressing treatment-related challenges, such as bacteriophage delivery, off-target effects, and bacterial resistance.

## 4 Optimizing formulation and encapsulation strategies for phage-based therapeutics

### 4.1 Ensuring the stability and activity

**Figure 3:**
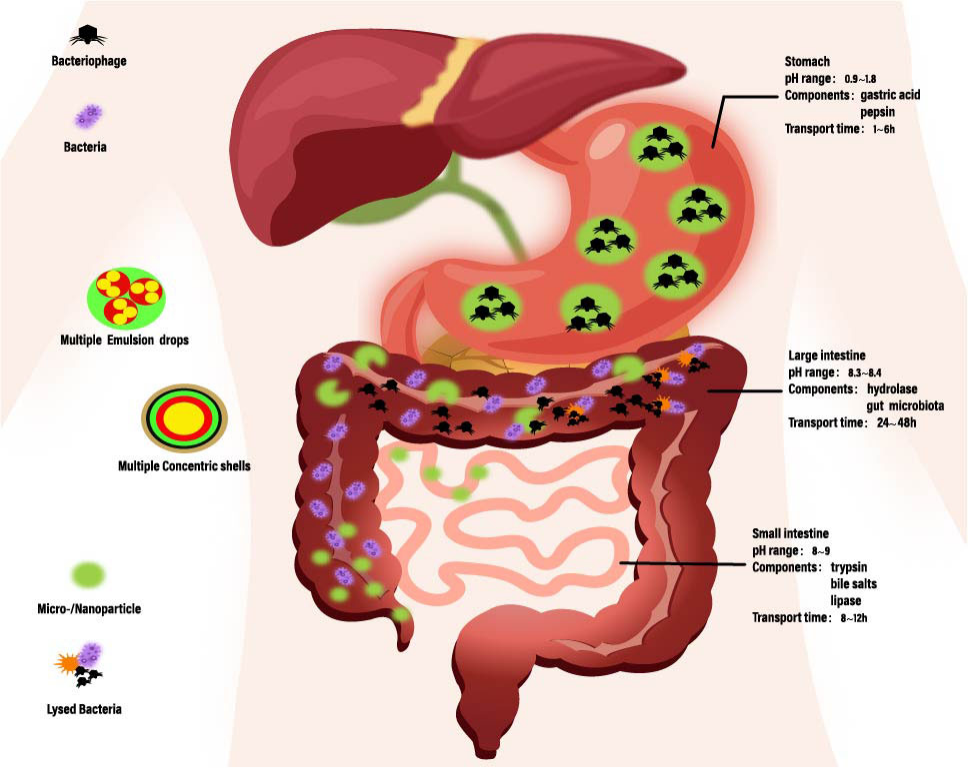
The pH range, composition of digestive fluids, and transit times across different segments of the gastrointestinal tract. Encapsulation within micro-/nanoparticles or stimulus-responsive systems allows targeted triggering or sustained release at specific sites. The encapsulation of multiple emulsion drops/multiple concentric shells also extends the circulation time of bacteriophages. [Adapted from (110)].

When developing bacteriophage therapy formulations, it is essential to understand the chemical and physical stresses that bacteriophages may undergo during preparation, processing, and storage to ensure their stability and activity. Bacteriophages consist mainly of proteins and are therefore susceptible to various factors that induce protein denaturation, such as types of organic solvents, high temperatures, pH values, ion concentrations, and interface effects. Mechanical pressures that may arise during formulation or encapsulation processes, such as shear forces during mixing, atomization during spraying, and stress during drying, also need to be carefully considered. Ensuring accurate dispersion of each bacteriophage particle is crucial during the development of formulations, although achieving complete dispersion poses significant challenges, certain techniques can achieve optimal results. Moreover, attention should be paid to controlling the morphology of bacteriophage particles to prevent aggregation or uncontrolled fusion with surrounding materials. Given the purpose and route of bacteriophage delivery, materials used to manufacture delivery vehicles should be safe, non-toxic, biocompatible, biodegradable, and non-irritating to the human body (108, 109). Additionally, encapsulation materials should have good resistance to various adverse environments. Furthermore, the residence time of ingested substances in the gastrointestinal tract is limited (**Figure 3**). Beyond the limited transport time, even if these encapsulated bacteriophages resist adverse conditions and are transported to the colon with high vitality, they will be excreted in the feces, resulting in minimal or no release of bacteriophages into the colon. Therefore, complete release of encapsulated bacteriophages is required when they are transported in the colon (**Table 3**).

**Table 3:** Overview of encapsulation materials and carriers for phage delivery.

Encapsulation materials/ Carrier systems	Advantages	Inherent limitations	Recent research advancements
Fiber	High stability; Controllable release rate	Limited applicability due to specific constraints; Challenges remain in reducing bacteriophage loss in manufacturing	Development of novel fiber materials
Emulsion	Maintaining high viability; Increased bioavailability; Delivery capability	Subject to temperature influence; Increased risk of bacterial contamination	Improvement in temperature stability
Hydrogel	Maintaining activity	Further research needed	Enhancement of performance of novel hydrogels
Eudragit S1/ Sodium alginate hydrogel	Maintaining activity; Acid resistance	Potential for higher costs	Improvement in cost-effectiveness
Liposomes	Providing physical protection and shielding from immune clearance; Prolonged retention time in the intestine	Difficult to maintain long-term stability	Optimization of transportation and storage stability
Particle materials	Adding protectants to increase survival rates; Higher stability observed across multiple formulation forms	Potentially higher costs	Reduction of production time

### 4.2 Enzyme/pH-responsive materials

Selection of enzymatically responsive materials capable of being degraded by specific enzymes produced by gut microbiota to release bacteriophages and pH-responsive materials that dissolve or swell under colonic pH conditions serves as ideal construction materials for achieving colonic-targeted delivery (111–114). Ahmad et al. prepared chitosan nanoparticles (CS-NP) and chitosan-phi KAZ14 bacteriophage-loaded nanoparticles (C-phi KAZ14 NP) using a simple entrapment method to effectively protect bacteriophages from gastric acid and enzymes in the chicken gastrointestinal tract. Chitosan nanoparticles exhibited potent protective effects on phi KAZ14 bacteriophages. Gel electrophoresis results demonstrated the protection of phi KAZ14 bacteriophages encapsulated in chitosan nanoparticles, while naked phi KAZ14 bacteriophages were degraded (115). *In vitro* release studies revealed complete inactivation of free bacteriophages under gastric conditions, while encapsulated bacteriophages maintained good vitality and steady release in the intestinal environment (9, 116).Pectin remains undigested in the stomach but can be degraded by pectinases secreted by intestinal bacteria, yielding short-chain fatty acids and simultaneously releasing encapsulated substances in the colon. Dini et al. demonstrated that emulsified low methoxylated pectin as a delivery system was more effective than unemulsified pectin, alginate, or emulsified alginate microspheres. Free non-encapsulated bacteriophages exposed to an environment with a pH of 2.5 showed a 3.7-log unit reduction in titer after 10 minutes. Additionally, in the presence of 0.5 mg/ml pepsin at pH 2.5, bacteriophage titer decreased to undetectable levels within 10 minutes. When bacteriophages were encapsulated in emulsified low methoxylated pectin spheres, there was no significant reduction in titer after 3 hours under conditions with a pH of 2.5 and a pepsin concentration as high as 4.2 mg/ml. These results indicate that bacteriophages encapsulated in emulsified low methoxylated pectin are protected from the harsh gastric environment (117). The differences in acidity among the stomach, small intestine, and colon enable the possibility of delivering bacteriophages to target sites using pH-responsive materials (118). Alginate is a commonly used pH-responsive material for bacteriophage encapsulation. It forms insoluble alginate in gastric fluid (pH < 3.0), protecting internal bacteriophages from gastric acid and digestive enzymes. Dlamini et al. employed alginate-carrageenan microcapsules to protect genetically diverse bacteriophages of five Salmonella species from simulated gastrointestinal conditions. Their study demonstrated effective encapsulation (>95%) and maintenance of viability (>87%) of five genetically diverse bacteriophages representing three genera after exposure to simulated gastric conditions (pH 2, 3.2 mg/ml pepsin, 37°C, for 1 hour). All bacteriophages were easily released from microcapsules at pH 7.5 and exposure to simulated duodenal conditions (pH 7, 10 mg/ml pancreatin, 37°C) (119). Eudragit S100 has been widely used as an encapsulation material for targeted bacteriophage release in the colon (5). For instance, Vinner et al. microencapsulated a Salmonella-specific bacteriophage Felix O1 in a pH-responsive polymer formulation. They incorporated the pH-responsive copolymer methacrylic acid Eudragit^®^ S100 (10% (w/v)) and added the biopolymer sodium alginate to the formulation. Results showed excellent protection of Felix O1 encapsulated in the formulation containing 10% (w/v) ES100 and 1% (w/v) alginate when exposed to simulated gastric fluid (SGF) (pH 1, for 2 hours). In simulated intestinal fluid (SIF), encapsulated bacteriophages previously exposed to SGF (pH 1, for 2 hours) were released as the pH increased, indicating inhibition of bacterial growth during the logarithmic growth phase (120).

### 4.3 Fiber and emulsion materials

**Figure 4:**
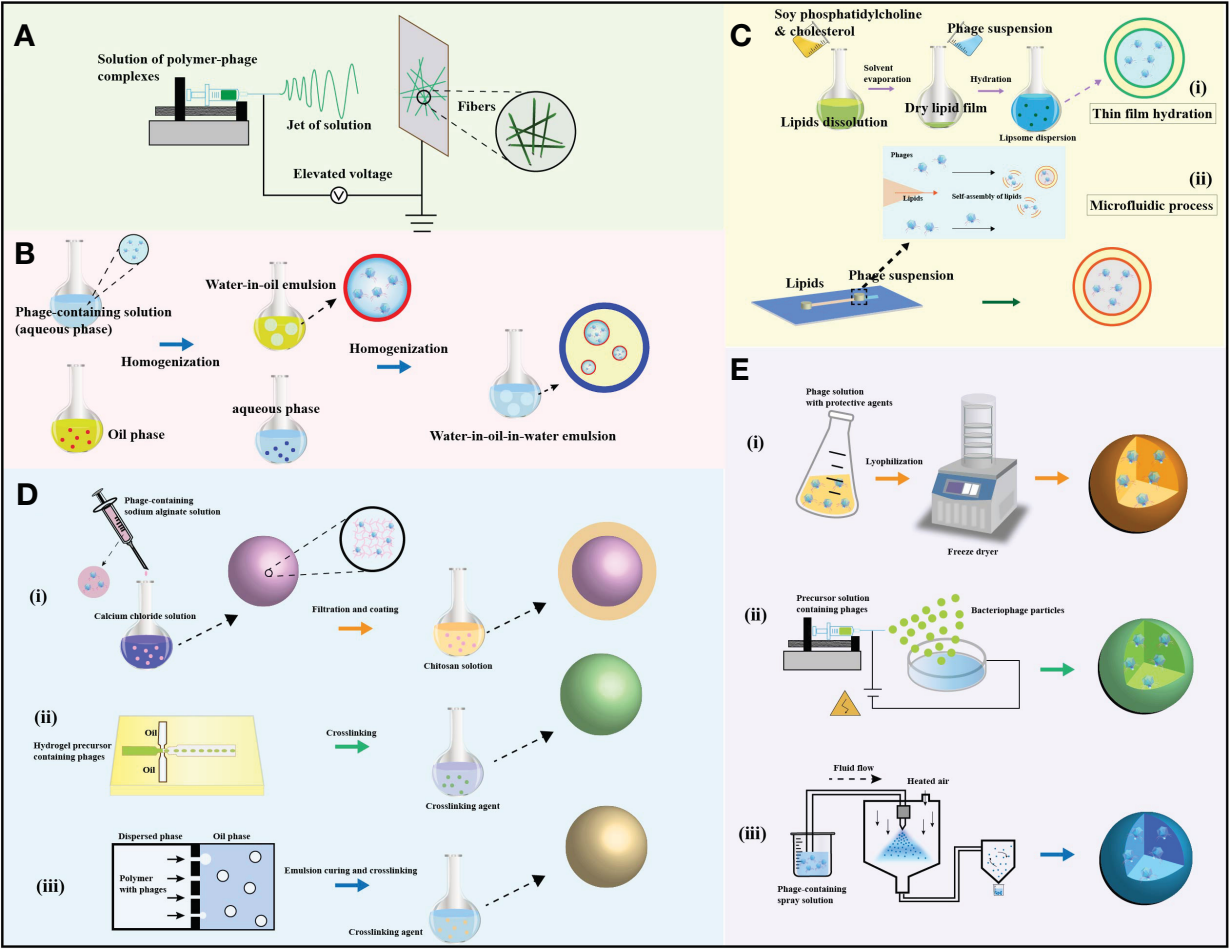
The preparation processes and microstructures of various carriers with encapsulated phages are outlined. **(A)** Depicts the preparation process of electrospun fibers and the microscopic structure of phages encapsulated through the electrospinning technique. **(B)** Illustrates the fabrication process and microstructure of multiple emulsions containing phages. **(C)** Presents a schematic of liposome preparation and the microscopic structures of encapsulated phages: (i) phages encapsulated in liposomes via the thin film hydration method, and (ii) phages in liposomes through a microfluidic process. **(D)** Describes the preparation mechanism and microstructure of hydrogels with encapsulated phages: (i) chitosan-coated alginate hydrogel loaded with phages using the extrusion-dripping method, (ii) Eudragit S100/alginate hydrogel with phages produced through a microfluidic technique, and (iii) Eudragit S100/alginate hydrogel with phages via membrane emulsification. **(E)** Provides a production diagram and microstructure of dry particles with phages: (i) phages in particles through lyophilization, (ii) dried particles with phages via the electrospraying process, and (iii) solid particles containing phages through the spray drying method. [Adapted from (82)].

Electrospinning technology is a versatile method for fiber production that does not require the use of toxic solvents or generate heat, providing a gentle encapsulation environment for bacteriophages (121, 122) (**Figure 4A**). For instance, Cheng et al. incorporated Escherichia coli bacteriophage T4 into poly(ϵ-caprolactone)/type I collagen (PCL-ColI) nanofibers using electrospinning to eradicate E. coli infections while establishing hemostatic functionality (123). To better maintain bacteriophage activity, R. korehei et al. pre-encapsulated T4 bacteriophages in an alginate hydrogel layer using an emulsification process and then incorporated them into fibers using electrospinning technology (124). The release of encapsulated bacteriophages from the fibers is mediated by the dissolution and/or swelling behavior of the fiber matrix (11). Thus, controlling the release rate of encapsulated bacteriophages from the fibers to the external environment can be achieved by altering the composition ratio of the composite fibers. Korehei et al. demonstrated that after injecting T4 bacteriophages into core/shell electrospun fibers prepared from poly(ethylene oxide) (PEO) and cellulose diacetate (CDA) or their mixtures, PEO-coated fibers released T4 bacteriophages immediately upon immersion in a buffer solution. The release rate of bacteriophages significantly decreased when CDA was mixed with PEO, and T4 bacteriophages were undetectable in fibers composed solely of CDA. Increasing the proportion of PEO in the fibers increased the diameter of the electrospun fibers and the viscosity of the release medium, resulting in a relatively slower release of T4 bacteriophages. The morphology of the electrospun fibers after release varied from discontinuous fibers to microexpanded fibers depending on the PEO/CDA ratio (11).

Encapsulation of bacteriophages in emulsions to form nanoparticles, followed by electrospinning, is another effective method to improve bacteriophage protection. Due to the hydrophilic nature of bacteriophages, W/O and W/O/W emulsions are commonly used for encapsulating bacteriophages (**Figure 4B**). For example, Puapermpoonsiri et al. demonstrated that selective bacteriophages against Staphylococcus aureus or Pseudomonas aeruginosa could be encapsulated into biodegradable polyester microspheres using an improved W/O/W solvent extraction scheme, with only partial loss of lytic activity (78). Following encapsulation, the aqueous core of the emulsion can provide a favorable environment for internal bacteriophages, promoting their survival during transportation and storage. Esteban et al. showed in their study the efficient encapsulation of bacteriophage K using water-in-oil nanoemulsions, demonstrating a novel method for the storage and delivery of bacteriophage K for the treatment of Staphylococcus aureus infections. The nanoemulsion-bacteriophage formulation became more stable and effective over time (81).

### 4.4 Liposomes and hydrogel materials

Liposomes, with their core containing an aqueous phase, offer a means to incorporate and encapsulate sensitive bacteriophages. Additionally, the phospholipid bilayer membrane of liposomes can provide physical protection for the core bacteriophages (**Figure 4C**). Cinquerrui et al. investigated the encapsulation of two model bacteriophages, preserving their activity and estimating the yield of microfluidic encapsulated bacteriophages (79). For certain intracellular diseases, liposomes are often employed to encapsulate bacteriophages for intracellular delivery to achieve therapeutic purposes. Nieth et al. found that liposome-encapsulated bacteriophages were more effectively absorbed into eukaryotic cells compared to free bacteriophages (80). Due to their excellent biocompatibility, liposomes show immense potential in protecting bacteriophages from immune clearance by preventing phagocytic cell recognition and capture. Researchers compared the ability of bacteriophages embedded in liposomes and free bacteriophages to enter mouse peritoneal macrophages and kill intracellular Klebsiella pneumoniae. The study also compared the efficacy of liposome-encapsulated bacteriophages alone or in combination with amikacin in eradicating mature biofilms. The results demonstrated that liposome-encapsulated bacteriophages were protected, capable of entering macrophages and killing intracellular bacteria. Liposome-encapsulated bacteriophages also exhibited synergistic activity with amikacin in biofilm eradication (125).

Hydrogels can serve as physical barriers for core bacteriophages, protecting them from acidic and enzymatic degradation while prolonging their retention time during intestinal transport, making them ideal carriers for bacteriophage delivery (**Figure 4D**). Kopac et al. proposed an efficient PolyHIPE hydrogel system for targeted bacteriophage delivery and rapid release at the target site. T7 bacteriophages were encapsulated in low cross-linked anionic nanofibrous cellulose hydrogels, which successfully protected the bacteriophages at pH below 3.9 (stomach), while the hydrogel network dissolved completely at pH above 3.9 (duodenum), allowing bacteriophage release. The PolyHIPE scaffold protected the hydrogel from mechanical stress during transport, preventing hydrogel collapse and accidental bacteriophage release (126). In constructing bacteriophage encapsulation carriers, a layer-by-layer assembly method was employed to coat acid-resistant polyethyleneimine and pectin onto alginate-bacteriophage hydrogels to protect bacteriophage activity during gastrointestinal transport. Hsu et al. generated alginate microbeads by dripping alginate solution into stirred calcium chloride solution, which were then coated with polyethyleneimine (PEI) and pectin. Film-coated alginate microbeads exhibited resistance to external pH changes. Increasing film thickness enhanced acid resistance. Encapsulating λ bacteriophages into alginate microbeads coated with (PEI/pectin) displayed excellent *in vitro* acid stability compared to free λ bacteriophages (127). Bacteriophage-based hydrogel-mediated delivery offers a precise strategy for modulating the expression of specific genes in individual microbes in the intestine, thereby promoting gut homeostasis and human health. The development of bacteriophage resistance is also a general consideration when delivering lytic bacteriophages (128). Therefore, the use of multiple bacteriophages (bacteriophage cocktails) may be necessary to ensure successful lytic activity and achieve the desired outcomes, broadening the host range of targeted microbes and suppressing the development of resistance (129).

### 4.5 Particle materials

The production methods for bacteriophage encapsulated particles mainly include freeze-drying, electrospraying, and spray-drying. Spray freeze-drying can produce controlled particle size distribution bacteriophage-loaded porous powders without subjecting bacteriophages to the high thermal stresses typically encountered in traditional spray-drying. Some protectants have been added to the formulation to enhance bacteriophage viability (**Figure 4E**). In Pereira et al.’s study, they developed an edible biopolymer microcapsule packaging for intestinal Salmonella, integrating lytic bacteriophage particles. For the formulation, a concentration of 2% (w/w) sodium alginate was added (130). To enhance colorectal cancer (CRC) chemotherapy effectiveness, nucleic acid bacteriophage-specific bacteriophages were conjugated with glucan nanoparticles loaded with CRC chemotherapeutic drugs to form bacteriophage-guided nanoparticles, which effectively inhibited nucleic acid bacteriophage growth and significantly extended the survival time of CRC mice (131). Additionally, in Thanki et al.’s study, combining polymers with sugars and leucine excipients also contributed to bacteriophage stability during drying (132). To precisely control bacteriophage dosage and improve oral administration convenience, these spray-dried particles can be further compressed into bacteriophage tablets for colonic delivery. In Khanal et al.’s study, targeting bacteriophage PEV20 against intestinal Pseudomonas aeruginosa, bacteriophage tablets suitable for oral administration were produced using industrial-scale tablet compression and coating machines. The bacteriophage tablet produced exhibited negligible reduction in bacteriophage titer throughout the process and passed the British Pharmacopoeia tests, including friability, weight variation, disintegration, and dissolution of uncoated tablets (in 0.1 M HCl and pH 7.4 phosphate buffer). The developed formulation method can be used to produce tablets containing other bacteriophages and bacteriophage cocktails, which are effective in combating intestinal bacterial infections (133).

## 5 Conclusion and outlook

Dysbiosis of the gut microbiota is linked with inflammatory bowel disease. Recently, bacteriophage delivery has emerged as an innovative strategy, holding immense potential to alter gut microbiota composition or modulate bacterial genes through their specificity for certain host bacteria. Bacteriophages play a pivotal role in the structure and functionality of human gut microbiota, thereby affecting gastrointestinal health and disease. Given this, bacteriophages stand out as promising agents for IBD therapy, targeting pathogenic gastrointestinal bacteria. However, environmental and gastrointestinal conditions can deactivate bacteriophages, diminishing their effectiveness and presenting substantial delivery challenges. Encapsulation techniques have been employed to boost bacteriophage vitality and stability for storage and intestinal transit. Various carriers have proven effective in preserving bacteriophage stability for storage, safeguarding viability, and ensuring efficient release in the colon during gastrointestinal passage. Despite some identified limitations with certain carriers, employing a combination of methods to create composite carriers presents research opportunities to enhance bacteriophage delivery in gut microbiota modulation practically.

## Author contributions

YL: Conceptualization, Data curation, Investigation, Methodology, Software, Supervision, Writing – original draft, Writing – review & editing. XL: Conceptualization, Investigation, Writing – original draft. HD: Writing – review & editing, Formal analysis, Resources, Software. KY: Data curation, Investigation, Software, Writing – review & editing. JY: Conceptualization, Investigation, Writing – original draft.

## References

[1] Wang Y He Y Liang Y Liu H Chen X Kulyar MF. Fecal microbiota transplantation attenuates Escherichia coli infected outgrowth by modulating the intestinal microbiome. Microb Cell Fact. (2023) 22:30. doi: 10.1186/s12934-023-02027-z 36803386 PMC9936653

[2] Awoniyi M Wang J Ngo B Meadows V Tam J Viswanathan A. Protective and aggressive bacterial subsets and metabolites modify hepatobiliary inflammation and fibrosis in a murine model of PSC. Gut. (2023) 72:671–68. doi: 10.1136/gutjnl-2021-326500 PMC9751228 35705368

[3] Zuo T Wong SH Lam K Lui R Cheung K Tang W. Bacteriophage transfer during faecal microbiota transplantation in Clostridium difficile infection is associated with treatment outcome. Gut. (2018) 67:634–43. doi: 10.1136/gutjnl-2017-313952 PMC5868238 28539351

[4] Kiss K Biri-Kovács B Szabó R Randelovic I Enyedi KN Schlosser G. Sequence modification of heptapeptide selected by phage display as homing device for HT-29 colon cancer cells to improve the anti-tumour activity of drug delivery systems. Eur J Medicinal Chem. (2019) 176:105–16. doi: 10.1016/j.ejmech.2019.05.016 31100648

[5] Tabare E Dauchot T Cochez C Glonti T Antoine C Laforêt F. Eudragit® FS microparticles containing bacteriophages, prepared by spray-drying for oral administration. Pharmaceutics. (2023) 15. doi: 10.3390/pharmaceutics15061602 PMC10305712 37376051

[6] Kuźmińska-Bajor M Śliwka P Korzeniowski P Kuczkowski M Moreno DS Woźniak-Biel A. Effective reduction of Salmonella Enteritidis in broiler chickens using the UPWr_S134 phage cocktail. Front Microbiol. (2023) 14:1136261. doi: 10.3389/fmicb.2023.1136261 37180264 PMC10174237

[7] Majewska J Miernikiewicz P Szymczak A Goszczyński TM Owczarek B Rybicka I. Evolution of the T4 phage virion is driven by selection pressure from non-bacterial factors. Microbiol Spectr. (2023) 11:e0011523. doi: 10.1128/spectrum.00115-23 37724862 PMC10580926

[8] Zalewska-Piątek B. Phage therapy-challenges, opportunities and future prospects. Pharm (Basel). (2023) 16. doi: 10.3390/ph16121638 PMC10747886 38139765

[9] Ma Y Pacan JC Wang Q Xu Y Huang X Korenevsky A. Microencapsulation of bacteriophage felix O1 into chitosan-alginate microspheres for oral delivery. Appl Environ Microbiol. (2008) 74:4799–805. doi: 10.1128/aem.00246-08 PMC2519356 18515488

[10] Khan KJ Dubinsky MC Ford AC Ullman TA Talley NJ Moayyedi P. Efficacy of immunosuppressive therapy for inflammatory bowel disease: a systematic review and meta-analysis. Am J Gastroenterol. (2011) 106:630–42. doi: 10.1038/ajg.2011.64 21407186

[11] Korehei R Kadla JF. Encapsulation of T4 *bacteriophage* in electrospun poly(ethylene oxide)/cellulose diacetate fibers. Carbohydr Polymers. (2014) 100:150–7. doi: 10.1016/j.carbpol.2013.03.079 24188849

[12] Li H Wang Y Shao S Yu H Wang D Li C. Rabdosia serra alleviates dextran sulfate sodium salt-induced colitis in mice through anti-inflammation, regulating Th17/Treg balance, maintaining intestinal barrier integrity, and modulating gut microbiota. J Pharm Anal. (2022) 12:824–38. doi: 10.1016/j.jpha.2022.08.001 PMC9805946 36605573

[13] Simmonds NJ Millar AD Blake DR Rampton DS. Antioxidant effects of aminosalicylates and potential new drugs for inflammatory bowel disease: assessment in cell-free systems and inflamed human colorectal biopsies. Aliment Pharmacol Ther. (1999) 13:363–72. doi: 10.1046/j.1365-2036.1999.00484.x 10102970

[14] Kaiser GC Yan F Polk DB. Mesalamine blocks tumor necrosis factor growth inhibition and nuclear factor κB activation in mouse colonocytes. Gastroenterology. (1999) 116:602–9. doi: 10.1016/s0016-5085(99)70182-4 PMC3606885 10029619

[15] Schwab M Reynders V Loitsch S Shastri YM Steinhilber D Schröder O. PPARγ is involved in mesalazine-mediated induction of apoptosis and inhibition of cell growth in colon cancer cells. Carcinogenesis. (2008) 29:1407–14. doi: 10.1093/carcin/bgn118 18544567

[16] Bersuder E Terciolo C Lechevrel M Martin E Quesnelle C Freund JN. Mesalazine initiates an anti-oncogenic β-catenin / MUCDHL negative feed-back loop in colon cancer cells by cell-specific mechanisms. BioMed Pharmacother. (2022) 146:112543. doi: 10.1016/j.biopha.2021.112543 34929577

[17] Somsouk M Dunham RM Cohen M Albright R Abdel-Mohsen M Liegler T. The immunologic effects of mesalamine in treated HIV-infected individuals with incomplete CD4+ T cell recovery: a randomized crossover trial. PloS One. (2014) 9:e116306. doi: 10.1371/journal.pone.0116306 25545673 PMC4283685

[18] Simpson JB Sekela JJ Carry BS Beaty V Patel S Redinbo MR. Diverse but desolate landscape of gut microbial azoreductases: A rationale for idiopathic IBD drug response. Gut Microbes. (2023) 15:2203963. doi: 10.1080/19490976.2023.2203963 37122075 PMC10132220

[19] Chang SS Hu HY. Long-term use of steroids protects from the development of symptomatic diverticulitis requiring hospitalization in the Asian population. PloS One. (2015) 10:e0124598. doi: 10.1371/journal.pone.0124598 25919040 PMC4412717

[20] Sperger J Shah KS Lu M Zhang X Ungaro RC Brenner EJ. Development and validation of multivariable prediction models for adverse COVID-19 outcomes in patients with IBD. BMJ Open. (2021) 11:e049740. doi: 10.1136/bmjopen-2021-049740 PMC8593277 34772750

[21] Seamons A Haenisch M Meeker S Pershutkina O Brabb T Treuting PM. Protective effects of ALDH1A enzyme inhibition on helicobacter-induced colitis in Smad3(-/-) mice are associated with altered α4ß7 integrin expression on activated T cells. Nutrients. doi: 10.3390/nu12102927 PMC7599670 32987910

[22] Jiang X Luo X Nan Q Ye Y Miao Y Miao J. Application of deep learning in the diagnosis and evaluation of ulcerative colitis disease severity. Therap Adv Gastroenterol. (2023) 16:17562848231215579. doi: 10.1177/17562848231215579 PMC10748675 38144424

[23] Adams SM Bornemann PH. Ulcerative colitis. Am Fam Physician. (2013) 87:699–705. 23939448

[24] Han KH Park JM Jeong M Han YM Go EJ Park J. Heme Oxygenase-1 Induction and Anti-inflammatory Actions of Atractylodes macrocephala and Taraxacum herba Extracts Prevented Colitis and Was More Effective than Sulfasalazine in Preventing Relapse. Gut Liver. (2017) 11:655–66. doi: 10.5009/gnl16496 PMC5593328 28651306

[25] Alameddine Z Abi Melhem R Dimachkie R Rabah H Chehab H El Khoury M. Risk of nephrolithiasis in patients with inflammatory bowel disease receiving biologic treatment. J Clin Med. (2023) 12. doi: 10.3390/jcm12196114 PMC10573832 37834757

[26] Ungar B Levy I Yavne Y Yavzori M Picard O Fudim E. Optimizing anti-TNF-α Therapy: serum levels of infliximab and adalimumab are associated with mucosal healing in patients with inflammatory bowel diseases. Clin Gastroenterol Hepatol. (2016) 14:550–7. doi: 10.1016/j.cgh.2015.10.025 26538204

[27] Herrlinger KR Stange EF. Infliximab, azathioprine, or combination therapy for Crohn's disease. New Engl J Med. (2010) 363:1086–6. doi: 10.1056/NEJMc1005805 20825325

[28] Berends SE D'Haens G Schaap T de Vries A Rispens T Bloem K. Dried blood samples can support monitoring of infliximab concentrations in patients with inflammatory bowel disease: A clinical validation. Br J Clin Pharmacol. (2019) 85:1544–51. doi: 10.1111/bcp.13939 PMC6595298 30927375

[29] Sandborn WJ van Assche G Reinisch W Colombel JF D'Haens G Wolf DC. Adalimumab induces and maintains clinical remission in patients with moderate-to-severe ulcerative colitis. Gastroenterology. (2012) 142:257–+. doi: 10.1053/j.gastro.2011.10.032 22062358

[30] Sandborn WJ Feagan BG Marano C Zhang HY Strauss R Johanns J. Subcutaneous golimumab induces clinical response and remission in patients with moderate-to-severe ulcerative colitis. Gastroenterology. (2014) 146:85–95. doi: 10.1053/j.gastro.2013.05.048 23735746

[31] Neurath MF. COVID-19 and immunomodulation in IBD. Gut. (2020) 69:1335–42. doi: 10.1136/gutjnl-2020-321269 PMC7211083 32303609

[32] Heidbreder K Sommer K Wiendl M Müller TM Atreya I Hildner K. Nr4a1-dependent non-classical monocytes are important for macrophage-mediated wound healing in the large intestine. Front Immunol. (2022) 13:1040775. doi: 10.3389/fimmu.2022.1040775 36741412 PMC9890957

[33] Fischer A Zundler S Atreya R Rath T Voskens C Hirschmann S. Differential effects of α4β7 and GPR15 on homing of effector and regulatory T cells from patients with UC to the inflamed gut in *vivo* . Gut. (2016) 65:1642–U290. doi: 10.1136/gutjnl-2015-310022 26209553 PMC5036234

[34] Zhang J Liu MH Gao X Dong C Li YX. Vedolizumab-associated diffuse interstitial lung disease in patients with ulcerative colitis: A case report. World J Clin Cases. (2022) 10:1716–22. doi: 10.12998/wjcc.v10.i5.1716 PMC8855246 35211614

[35] Asscher VER Biemans VBC Pierik MJ Dijkstra G Löwenberg M van der Marel S. Comorbidity, not patient age, is associated with impaired safety outcomes in vedolizumab- and ustekinumab-treated patients with inflammatory bowel disease-a prospective multicentre cohort study. Aliment Pharmacol Ther. (2020) 52:1366–76. doi: 10.1111/apt.16073 PMC7539998 32901983

[36] Sandborn WJ Gasink C Gao LL Blank MA Johanns J Guzzo C. Ustekinumab induction and maintenance therapy in refractory Crohn's disease. New Engl J Med. (2012) 367:1519–28. doi: 10.1056/NEJMoa1203572 23075178

[37] Mannon PJ Fuss IJ Mayer L Elson CO Sandborn WJ Present D. Anti ILCsDSG. Anti-interleukin-12 antibody for active Crohn's disease. New Engl J Med. (2004) 351:2069–79. doi: 10.1056/NEJMoa033402 15537905

[38] Yen D Cheung J Scheerens H Poulet F McClanahan T McKenzie B. IL-23 is essential for T cell-mediated colitis and promotes inflammation *via* IL-17 and IL-6. J Clin Invest. (2006) 116:1310–6. doi: 10.1172/jci21404 PMC1451201 16670770

[39] Jacob N Jacobs JP Kumagai K Ha CWY Kanazawa Y Lagishetty V. Inflammation-independent TL1A-mediated intestinal fibrosis is dependent on the gut microbiome. Mucosal Immunol. (2018) 11:1466–76. doi: 10.1038/s41385-018-0055-y PMC6162160 29988118

[40] Clarke AW Poulton L Shim D Mabon D Butt D Pollard M. An anti-TL1A antibody for the treatment of asthma and inflammatory bowel disease. Mabs. (2018) 10:664–77. doi: 10.1080/19420862.2018.1440164 PMC5973687 29436901

[41] Shih DQ Zheng L Zhang X Zhang H Kanazawa Y Ichikawa R. Inhibition of a novel fibrogenic factor Tl1a reverses established colonic fibrosis. Mucosal Immunol. (2014) 7:1492–503. doi: 10.1038/mi.2014.37 PMC4205266 24850426

[42] Liang J Sha SM Wu KC. Role of the intestinal microbiota and fecal transplantation in inflammatory bowel diseases. J Digest Dis. (2014) 15:641–6. doi: 10.1111/1751-2980.12211 25389085

[43] Eiseman B Silen W Bascom GS Kauvar AJ. Fecal enema as an adjunct in the treatment of pseudomembranous enterocolitis. Surgery. (1958) 44:854–9. 13592638

[44] Khan I Ullah N Zha L Bai Y Khan A Zhao T. Alteration of gut microbiota in inflammatory bowel disease (IBD): cause or consequence? IBD treatment targeting the gut microbiome. Pathogens. (2019) 8. doi: 10.3390/pathogens8030126 PMC6789542 31412603

[45] Guarner F Malagelada JR. Gut flora in health and disease. Lancet. (2003) 361:512–9. doi: 10.1016/s0140-6736(03)12489-0 12583961

[46] Cananzi M Wohler E Marzollo A Colavito D You J Jing H. IFIH1 loss-of-function variants contribute to very early-onset inflammatory bowel disease. Hum Genet. (2021) 140:1299–312. doi: 10.1007/s00439-021-02300-4 PMC8423350 34185153

[47] Amoroso C Perillo F Strati F Fantini MC Caprioli F Facciotti F. The role of gut microbiota biomodulators on mucosal immunity and intestinal inflammation. Cells. (2020) 9. doi: 10.3390/cells9051234 PMC7291275 32429359

[48] Moayyedi P Surette MG Kim PT Libertucci J Wolfe M Onischi C. Fecal microbiota transplantation induces remission in patients with active ulcerative colitis in a randomized controlled trial. Gastroenterology. (2015) 149:102–+. doi: 10.1053/j.gastro.2015.04.001 25857665

[49] Paramsothy S Kamm MA Kaakoush NO Walsh AJ van den Bogaerde J Samuel D. Multidonor intensive faecal microbiota transplantation for active ulcerative colitis: a randomised placebo-controlled trial. Lancet. (2017) 389:1218–28. doi: 10.1016/s0140-6736(17)30182-4 28214091

[50] Costello SP Hughes PA Waters O Bryant RV Vincent AD Blatchford P. Effect of fecal microbiota transplantation on 8-week remission in patients with ulcerative colitis A randomized clinical trial. Jama Journal Am Med Assoc. (2019) 321:156–64. doi: 10.1001/jama.2018.20046 PMC6439766 30644982

[51] Chen HT Huang HL Xu HM Luo QL He J Li YQ. Fecal microbiota transplantation ameliorates active ulcerative colitis. Exp Ther Med. (2020) 19:2650–60. doi: 10.3892/etm.2020.8512 PMC7086197 32256746

[52] Paramsothy S Paramsothy R Rubin DT Kamm MA Kaakoush NO Mitchell HM. Faecal microbiota transplantation for inflammatory bowel disease: A systematic review and meta-analysis. J Crohns Colitis. (2017) 11:1180–99. doi: 10.1093/ecco-jcc/jjx063 28486648

[53] Chen T Zhou Q Zhang D Jiang F Wu J Zhou JY. Effect of faecal microbiota transplantation for treatment of *Clostridium difficile* infection in patients with inflammatory bowel disease: A systematic review and meta-analysis of cohort studies. J Crohns Colitis. (2018) 12:710–7. doi: 10.1093/ecco-jcc/jjy031 29528385

[54] Qiu P Ishimoto T Fu LF Zhang J Zhang ZY Liu Y. The gut microbiota in inflammatory bowel disease. Front Cell Infect Microbiol. (2022) 12:733992. doi: 10.3389/fcimb.2022.733992 35273921 PMC8902753

[55] Mirsepasi-Lauridsen HC Vallance BA Krogfelt KA Petersen AM. *Escherichia coli* pathobionts associated with inflammatory bowel disease. Clin Microbiol Rev. (2019) 32. doi: 10.1128/cmr.00060-18 PMC6431131 30700431

[56] Glassner KL Abraham BP Quigley EMM. The microbiome and inflammatory bowel disease. J Allergy Clin Immunol. (2020) 145:16–27. doi: 10.1016/j.jaci.2019.11.003 31910984

[57] Thia KT Mahadevan U Feagan BG Wong C Cockeram A Bitton A. Ciprofloxacin or metronidazole for the treatment of perianal fistulas in patients with Crohn's disease: A randomized, double-blind, placebo-controlled pilot study. Inflamm Bowel Dis. (2009) 15:17–24. doi: 10.1002/ibd.20608 18668682

[58] Lahat G Halperin D Barazovsky E Shalit I Rabau M Klausner J. Immunomodulatory effects of ciprofloxacin in TNBS-induced colitis in mice. Inflamm Bowel Dis. (2007) 13:557–65. doi: 10.1002/ibd.20077 17253612

[59] Ianiro G Tilg H Gasbarrini A. Antibiotics as deep modulators of gut microbiota: between good and evil. Gut. (2016) 65:1906–15. doi: 10.1136/gutjnl-2016-312297 27531828

[60] Rutgeerts P Hiele M Geboes K Peeters M Penninckx F Aerts R. Controlled trial of metronidazole treatment for prevention of Crohn's recurrence after ileal resection. Gastroenterol 1995. (1995) 108:1617–21. doi: 10.1016/0016-5085(95)90121-3 7768364

[61] Prantera C Berto E Scribano ML Falasco G. Use of antibiotics in the treatment of active Crohn's disease: experience with metronidazole and ciprofloxacin. Ital J Gastroenterol hepatology. 1998. (1998) 30:602–6. 10076781

[62] Recharla N Geesala R Shi XZ. Gut microbial metabolite butyrate and its therapeutic role in inflammatory bowel disease: A literature review. Nutrients. (2023) 15. doi: 10.3390/nu15102275 PMC10221771 37242159

[63] Chelakkot C Ghim J Ryu SH. Mechanisms regulating intestinal barrier integrity and its pathological implications. Exp Mol Med. (2018) 50:1–9. doi: 10.1038/s12276-018-0126-x PMC6095905 30115904

[64] Henderson P van Limbergen JE Schwarze J Wilson DC. Function of the intestinal epithelium and its dysregulation in inflammatory bowel disease. Inflammation Bowel Dis. (2011) 17:382–95. doi: 10.1002/ibd.21379 20645321

[65] Górski A Jończyk-Matysiak E Łusiak-Szelachowska M Międzybrodzki R Weber-Dąbrowska B Borysowski J. Bacteriophages targeting intestinal epithelial cells: a potential novel form of immunotherapy. Cell Mol Life Sci. (2018) 75:589–95. doi: 10.1007/s00018-017-2715-6 PMC5769817 29164271

[66] Xie C Quan R Hong F Zou K Yan W Fu Y. The culprit of mesalamine intolerance: case series and literature review. BMC Gastroenterol. (2019) 19:138. doi: 10.1186/s12876-019-1049-2 31366329 PMC6670194

[67] Huang C Hao W Wang X Zhou R Lin Q. Probiotics for the treatment of ulcerative colitis: a review of experimental research from 2018 to 2022. Front Microbiol. (2023) 14:1211271. doi: 10.3389/fmicb.2023.1211271 37485519 PMC10358780

[68] Abdallah HMI Ammar NM Abdelhameed MF Gendy A Ragab TIM Abd-ElGawad AM. Protective Mechanism of Acacia saligna Butanol Extract and Its Nano-Formulations against Ulcerative Colitis in Rats as Revealed *via* Biochemical and Metabolomic Assays. Biol (Basel). (2020) 9. doi: 10.3390/biology9080195 PMC7463518 32751448

[69] Fukuda K Fujita Y. Determination of the discriminant score of intestinal microbiota as a biomarker of disease activity in patients with ulcerative colitis. BMC Gastroenterol. (2014) 14. doi: 10.1186/1471-230x-14-49 PMC3999879 24641276

[70] Mishra J Stubbs M Kuang L Vara N Kumar P Kumar N. Inflammatory bowel disease therapeutics: A focus on probiotic engineering. Mediators Inflamm. (2022) 2022:9621668. doi: 10.1155/2022/9621668 35082553 PMC8786545

[71] Li S Xu K Cheng Y Chen L Yi A Xiao Z. The role of complex interactions between the intestinal flora and host in regulating intestinal homeostasis and inflammatory bowel disease. Front Microbiol. (2023) 14:1188455. doi: 10.3389/fmicb.2023.1188455 37389342 PMC10303177

[72] Ohkusa T Okayasu I Ogihara T Morita K Ogawa M Sato N. Induction of experimental ulcerative colitis by Fusobacterium varium isolated from colonic mucosa of patients with ulcerative colitis. Gut. (2003) 52:79–83. doi: 10.1136/gut.52.1.79 12477765 PMC1773498

[73] Federici S Kviatcovsky D Valdés-Mas R Elinav E. Microbiome-phage interactions in inflammatory bowel disease. Clin Microbiol Infect. (2023) 29:682–8. doi: 10.1016/j.cmi.2022.08.027 36191844

[74] Federici S Kredo-Russo S Valdés-Mas R Kviatcovsky D Weinstock E Matiuhin Y. Targeted suppression of human IBD-associated gut microbiota commensals by phage consortia for treatment of intestinal inflammation. Cell. (2022) 185:2879–+. doi: 10.1016/j.cell.2022.07.003 35931020

[75] Sinha A Li Y Mirzaei MK Shamash M Samadfam R King IL. Transplantation of bacteriophages from ulcerative colitis patients shifts the gut bacteriome and exacerbates the severity of DSS colitis. Microbiome. (2022) 10. doi: 10.1186/s40168-022-01275-2 PMC9264660 35799219

[76] Titécat M Rousseaux C Dubuquoy C Foligné B Rahmouni O Mahieux S. Safety and efficacy of an AIEC-targeted bacteriophage cocktail in a mice colitis model. J Crohns Colitis. (2022) 16:1617–27. doi: 10.1093/ecco-jcc/jjac064 35997152

[77] Duerkop BA Kleiner M Paez-Espino D Zhu WH Bushnell B Hassell B. Murine colitis reveals a disease-associated bacteriophage community. Nat Microbiol. (2018) 3:1023–31. doi: 10.1038/s41564-018-0210-y PMC6112176 30038310

[78] Puapermpoonsiri U Spencer J van der Walle CF. A freeze-dried formulation of bacteriophage encapsulated in biodegradable microspheres. Eur J Pharm Biopharm. (2009) 72:26–33. doi: 10.1016/j.ejpb.2008.12.001 19118627

[79] Cinquerrui S Mancuso F Vladisavljevic GT Bakker SE Malik DJ. Nanoencapsulation of bacteriophages in liposomes prepared using microfluidic hydrodynamic flow focusing. Front Microbiol. (2018), 9. doi: 10.3389/fmicb.2018.02172 30258426 PMC6144953

[80] Nieth A Verseux C Barnert S Süss R Römer W. A first step toward liposome-mediated intracellular bacteriophage therapy. Expert Opin Drug Deliv. (2015) 12:1411–24. doi: 10.1517/17425247.2015.1043125 25937143

[81] Esteban PP Alves DR Enright MC Bean JE Gaudion A Jenkins ATA. Enhancement of the Antimicrobial Properties of Bacteriophage-K *via* Stabilization using Oil-in-Water Nano-Emulsions. Biotechnol Progress. (2014) 30:932–44. doi: 10.1002/btpr.1898 24616404

[82] Yang YF Du H Zou G Song ZY Zhou Y Li H. Encapsulation and delivery of phage as a novel method for gut flora manipulation in *situ*: A review. J Controlled Release. (2023) 353:634–49. doi: 10.1016/j.jconrel.2022.11.048 36464065

[83] Ramires LC Santos GS Ramires RP da Fonseca LF Jeyaraman M Muthu S. The association between gut microbiota and osteoarthritis: does the disease begin in the gut? Int J Mol Sci. (2022) 23. doi: 10.3390/ijms23031494 PMC8835947 35163417

[84] Lv HH Tao FY Peng LL Chen SF Ren ZY Chen JH. *In Vitro* Probiotic Properties of *Bifidobacterium animalis* subsp. *lactis* SF and Its Alleviating Effect on Non-Alcoholic Fatty Liver Disease. Nutrients. (2023) 15. doi: 10.3390/nu15061355 PMC10053994 36986084

[85] Gundogdu A Bolkvadze D Kilic H. *In vitro* Effectiveness of Commercial Bacteriophage Cocktails on Diverse Extended-Spectrum Beta-Lactamase Producing Escherichia coli Strains. Front Microbiol. (2016) 7:1761. doi: 10.3389/fmicb.2016.01761 27857711 PMC5093111

[86] Shuwen H Kefeng D. Intestinal phages interact with bacteria and are involved in human diseases. Gut Microbes. (2022) 14:2113717. doi: 10.1080/19490976.2022.2113717 36037202 PMC9427043

[87] Allué-Guardia A Saranathan R Chan J Torrelles JB. Mycobacteriophages as potential therapeutic agents against drug-resistant tuberculosis. Int J Mol Sci. (2021) 22. doi: 10.3390/ijms22020735 PMC7828454 33450990

[88] Luo Q Liu N Pu S Zhuang Z Gong H Zhang D. A review on the research progress on non-pharmacological therapy of Helicobacter pylori. Front Microbiol. (2023) 14:1134254. doi: 10.3389/fmicb.2023.1134254 37007498 PMC10063898

[89] Ul Haq I Krukiewicz K Yahya G Haq MU Maryam S Mosbah RA. The breadth of bacteriophages contributing to the development of the phage-based vaccines for COVID-19: an ideal platform to design the multiplex vaccine. Int J Mol Sci. (2023). doi: 10.3390/ijms24021536 PMC9861788 36675046

[90] Yao Q Wu C Yu X Chen X Pan G Chen B. Current material engineering strategies to prevent catheter encrustation in urinary tracts. Mater Today Bio. (2022) 16:100413. doi: 10.1016/j.mtbio.2022.100413 PMC9474921 36118951

[91] Silveira CB Rohwer FL. Piggyback-the-Winner in host-associated microbial communities. NPJ Biofilms Microb. (2016) 2:16010. doi: 10.1038/npjbiofilms.2016.10 PMC5515262 28721247

[92] Liang X Radosevich M. Commentary: A host-produced quorum-sensing autoinducer controls a phage lysis-lysogeny decision. Front Microbiol. (2019) 10:1201. doi: 10.3389/fmicb.2019.01201 31231325 PMC6558226

[93] Smith MW Herfort L Rivers AR Simon HM. Genomic signatures for sedimentary microbial utilization of phytoplankton detritus in a fast-flowing estuary. Front Microbiol. (2019) 10:2475. doi: 10.3389/fmicb.2019.02475 31749780 PMC6848030

[94] Ko CC Hatfull GF. Mycobacteriophage Fruitloop gp52 inactivates Wag31 (DivIVA) to prevent heterotypic superinfection. Mol Microbiol. (2018) 108:443–60. doi: 10.1111/mmi.13946 PMC5943086 29488662

[95] Khan Mirzaei M Deng L. New technologies for developing phage-based tools to manipulate the human microbiome. Trends Microbiol. (2022) 30:131–42. doi: 10.1016/j.tim.2021.04.007 34016512

[96] Casey E McDonnell B White K Stamou P Crowley T O'Neill I. Needle in a whey-stack: PhRACS as a discovery tool for unknown phage-host combinations. mBio. (2022) 13:e0333421. doi: 10.1128/mbio.03334-21 35089052 PMC8725590

[97] Agarwal R Johnson CT Imhoff BR Donlan RM McCarty NA García AJ. Inhaled bacteriophage-loaded polymeric microparticles ameliorate acute lung infections. Nat BioMed Eng. (2018) 2:841–9. doi: 10.1038/s41551-018-0263-5 PMC6408147 30854250

[98] Teng F Xiong X Zhang S Li G Wang R Zhang L. Efficacy assessment of phage therapy in treating Staphylococcus aureus-induced mastitis in mice. Viruses. (2022) 14. doi: 10.3390/v14030620 PMC8954217 35337027

[99] Whon TW Kim HS Shin NR Sung H Kim MS Kim JY. Calf diarrhea caused by prolonged expansion of autochthonous gut enterobacteriaceae and their lytic bacteriophages. Msystems. doi: 10.1128/mSystems.00816-20 PMC8546982 33653940

[100] Hu J Ye H Wang S Wang J Han D. Prophage activation in the intestine: insights into functions and possible applications. Front Microbiol. (2021) 12:785634. doi: 10.3389/fmicb.2021.785634 34966370 PMC8710666

[101] Li J Qu W Hu C Liu Z Yan H. Antidepressants amitriptyline, fluoxetine, and traditional Chinese medicine Xiaoyaosan caused alterations in gut DNA virome composition and function in rats exposed chronic unpredictable mild stress. Front Microbiol. (2023) 14:1132403. doi: 10.3389/fmicb.2023.1132403 37125190 PMC10140408

[102] León Y Faherty CS. Bacteriophages against enteropathogens: rediscovery and refinement of novel antimicrobial therapeutics. Curr Opin Infect Dis. (2021) 34:491–9. doi: 10.1097/qco.0000000000000772 PMC8447223 34524200

[103] Huang D Yu P Ye M Schwarz C Jiang X Alvarez PJJ. Enhanced mutualistic symbiosis between soil phages and bacteria with elevated chromium-induced environmental stress. Microbiome. (2021) 9:150. doi: 10.1186/s40168-021-01074-1 34183048 PMC8240259

[104] Park JY Moon BY Park JW Thornton JA Park YH Seo KS. Genetic engineering of a temperate phage-based delivery system for CRISPR/Cas9 antimicrobials against Staphylococcus aureus. Sci Rep. (2017) 7:44929. doi: 10.1038/srep44929 28322317 PMC5359561

[105] Zia S Alkheraije KA. Recent trends in the use of bacteriophages as replacement of antimicrobials against food-animal pathogens. Front Vet Sci. (2023) 10:1162465. doi: 10.3389/fvets.2023.1162465 37303721 PMC10247982

[106] Ramirez K Cazarez-Montoya C Lopez-Moreno HS Castro-Del Campo N. Bacteriophage cocktail for biocontrol of *Escherichia coli* O157:H7: Stability and potential allergenicity study. PloS One. (2018) 13. doi: 10.1371/journal.pone.0195023 PMC5953568 29763937

[107] Duyvejonck H Merabishvili M Vaneechoutte M de Soir S Wright R Friman VP. Evaluation of the stability of bacteriophages in different solutions suitable for the production of magistral preparations in Belgium. Viruses-Basel. (2021) 13. doi: 10.3390/v13050865 PMC8151234 34066841

[108] Kim HY Chang RYK Morales S Chan HK. Bacteriophage-delivering hydrogels: current progress in combating antibiotic resistant bacterial infection. Antibiotics-Basel. (2021) 10. doi: 10.3390/antibiotics10020130 PMC7911734 33572929

[109] Korel A Samokhin A Zemlyakova E Pestov A Blinova E Zelikman M. A carboxyethylchitosan gel cross-linked with glutaraldehyde as a candidate carrier for biomedical applications. Gels (Basel Switzerland). (2023) 9. doi: 10.3390/gels9090756 PMC10531016 37754437

[110] Malik DJ Sokolov IJ Vinner GK Mancuso F Cinquerrui S Vladisavljevic GT. Formulation, stabilisation and encapsulation of bacteriophage for phage therapy. Adv Colloid Interface Sci. (2017) 249:100–33. doi: 10.1016/j.cis.2017.05.014 28688779

[111] Salatin S Khosroushahi AY. Overviews on the cellular uptake mechanism of polysaccharide colloidal nanoparticles. J Cell Mol Med. (2017) 21:1668–86. doi: 10.1111/jcmm.13110 PMC5571529 28244656

[112] Zhang SF Langer R Traverso G. Nanoparticulate drug delivery systems targeting inflammation for treatment of inflammatory bowel disease. Nano Today. (2017) 16:82–96. doi: 10.1016/j.nantod.2017.08.006 31186671 PMC6557461

[113] Li J Ma LP Wang C Jiang PJ Cui PF Wang JH. Rationally designed oral DOX gels for colon-specific administration. Gels. (2022) 8. doi: 10.3390/gels8120759 PMC9777853 36547283

[114] Guan ZW Feng Q. Chitosan and chitooligosaccharide: the promising non-plant-derived prebiotics with multiple biological activities. Int J Mol Sci. (2022) 23. doi: 10.3390/ijms23126761 PMC9223384 35743209

[115] Ahmad KA Mohammed AS Abas F. Chitosan nanoparticles as carriers for the delivery of ΦKAZ14 bacteriophage for oral biological control of colibacillosis in chickens. Molecules. (2016) 21. doi: 10.3390/molecules21030256 PMC6273744 26985885

[116] Mao XY Wu YX Ma RW Li L Wang LP Tan YZ. Oral phage therapy with microencapsulated phage A221 against Escherichia coli infections in weaned piglets. BMC Vet Res. (2023) 19. doi: 10.1186/s12917-023-03724-y PMC10510151 37730566

[117] Dini C Islan GA de Urraza PJ Castro GR. Novel biopolymer matrices for microencapsulation of phages: enhanced protection against acidity and protease activity. Macromol Biosci. (2012) 12:1200–8. doi: 10.1002/mabi.201200109 22847825

[118] Cieplak T Soffer N Sulakvelidze A Nielsen DS. A bacteriophage cocktail targeting *Escherichia coli reduces E-coli* in simulated gut conditions, while preserving a non-targeted representative commensal normal microbiota. Gut Microbes. (2018) 9:391–9. doi: 10.1080/19490976.2018.1447291 PMC6219645 29517960

[119] Dlamini SB Gigante AM Hooton SPT Atterbury RJ. Efficacy of different encapsulation techniques on the viability and stability of diverse phage under simulated gastric conditions. Microorganisms. (2023) 11. doi: 10.3390/microorganisms11102389 PMC10608910 37894046

[120] Vinner GK Malik DJ. High precision microfluidic microencapsulation of bacteriophages for enteric delivery. Res Microbiol. (2018) 169:522–30. doi: 10.1016/j.resmic.2018.05.011 29886256

[121] Bhardwaj N Kundu SC. Electrospinning: A fascinating fiber fabrication technique. Biotechnol Adv. (2010) 28:325–47. doi: 10.1016/j.bioteChadv.2010.01.004 20100560

[122] Rosner D Clark J. Formulations for bacteriophage therapy and the potential uses of immobilization. Pharmaceuticals. (2021) 14. doi: 10.3390/ph14040359 PMC8069877 33924739

[123] Cheng WL Zhang ZY Xu RD Cai PP Kristensen P Chen ML. Incorporation of bacteriophages in polycaprolactone/collagen fibers for antibacterial hemostatic dual-function. J Biomed Mater Res Part B-Applied Biomater. (2018) 106:2588–95. doi: 10.1002/jbm.b.34075 29356329

[124] Korehei R Kadla J. Incorporation of T4 bacteriophage in electrospun fibres. J Appl Microbiol. (2013) 114:1425–34. doi: 10.1111/jam.12158 23445225

[125] Singla S Harjai K Katare OP Chhibber S. Encapsulation of bacteriophage in liposome accentuates its entry in to macrophage and shields it from neutralizing antibodies. PloS One. (2016) 11. doi: 10.1371/journal.pone.0153777 PMC4846161 27115154

[126] Kopac T Lisac A Mravljak R Rucigaj A Krajnc M Podgornik A. Bacteriophage delivery systems based on composite PolyHIPE/nanocellulose hydrogel particles. Polymers. (2021) 13. doi: 10.3390/polym13162648 PMC8401677 34451188

[127] Hsu BB Plant IN Lyon L Anastassacos FM Way JC Silver PA. *In situ* reprogramming of gut bacteria by oral delivery. Nat Commun. (2020) 11. doi: 10.1038/s41467-020-18614-2 PMC7538559 33024097

[128] Topka G Bloch S Nejman-Falenczyk B Gasior T Jurczak-Kurek A Necel A. Characterization of bacteriophage vB-EcoS-95, isolated from urban sewage and revealing extremely rapid lytic development. Front Microbiol. (2019) 9:3326. doi: 10.3389/fmicb.2018.03326 30697202 PMC6340994

[129] Amankwah S Abdella K Kassa T. Bacterial biofilm destruction: A focused review on the recent use of phage-based strategies with other antibiofilm agents. Nanotechnol Sci Appl. (2021) 14:161–77. doi: 10.2147/nsa.S325594 PMC8449863 34548785

[130] Pereira AO Barros NMA Guerrero BR Emencheta SC Baldo DA Oliveira JM Jr.. An Edible Biopolymeric Microcapsular Wrapping Integrating Lytic Bacteriophage Particles for *Salmonella enterica*: Potential for Integration into Poultry Feed. Antibiotics-Basel. (2023) 12. doi: 10.3390/antibiotics12060988 PMC10295387 37370307

[131] Zheng DW Dong X Pan P Chen KW Fan JX Cheng SX. Phage-guided modulation of the gut microbiota of mouse models of colorectal cancer augments their responses to chemotherapy. Nat Biomed Eng. (2019) 3:717–28. doi: 10.1038/s41551-019-0423-2 31332342

[132] Thanki AM Mignard G Atterbury RJ Barrow P Millard AD Clokie MRJ. Prophylactic delivery of a bacteriophage cocktail in feed significantly reduces salmonella colonization in pigs. Microbiol Spectr. (2022) 10. doi: 10.1128/spectrum.00422-22 PMC9241700 35579475

[133] Khanal D Chang RYK Hick C Morales S Chan HK. Enteric-coated bacteriophage tablets for oral administration against gastrointestinal infections. Int J Pharm. (2021) 609. doi: 10.1016/j.ijpharm.2021.121206 34673163

